# Reader Responses to Online Reporting of Tagged Bird Behavior

**DOI:** 10.3390/ani15142053

**Published:** 2025-07-11

**Authors:** Louise Hayward

**Affiliations:** Social and Political Sciences, Philosophy and Anthropology (SPSPA), University of Exeter, Exeter EX4 4QJ, UK; lh769@exeter.ac.uk

**Keywords:** public opinion, online comments, tagging, wildlife, birds

## Abstract

This paper explores responses to online coverage of an avian tracking project. Researchers attached novel trackers to a small group of wild magpies (*Gymnorhina tibicen*). These were subsequently removed by conspecifics, an example of ‘rescue behavior’ that was recounted in several media outlets. Online comments on three articles, from across the political spectrum (the *Conversation*, UK *Guardian*, and UK *Daily Mail*), were selected for thematic analysis. The resulting 680 comments were analyzed qualitatively and quantitatively to uncover predominant themes and the overall balance of positive and negative sentiments expressed about this tagging project or wildlife tagging generally. Topics occurring most frequently were themed into three interrelated areas: (1) sharing personal feelings and experiences, (2) comparing the merits of different species, and (3) sharing knowledge and opinion. Twenty-one percent (21%) of respondents expressed an opinion on the ethics of wildlife tagging. In the *Daily Mail* and *Guardian*, this opinion was more likely to be negative towards the use of tags. Opinion was more balanced for readers of the *Conversation*’s article. Willingness to comment on online news is low, and readers of this story were not asked directly for their opinion. Nevertheless, the data here illustrate some public perceptions of wildlife tagging, and there was a clear negative reaction from many responders. Widening the means through which people can engage with animal science has the potential to advance discussions around research ethics and animal welfare. Reactions to this story expose important questions for scientists seeking to engage with, and convince, the public of the merits of their work.

## Simple Summary

This paper explores responses to online reporting of an animal tracking research project. Researchers attached tracking devices to Australian Magpies (*Gymnorhina tibicen*) via a specially designed harness. The harnesses were quickly removed by other birds in the group, and this interesting behavior was reported on a number of news sites. Reported here is an analysis of readers’ reactions to this story, as expressed in online comment sections. The most common reasons for posting a comment were (1) sharing personal feelings and experiences, (2) comparing the merits of different species, and (3) sharing knowledge and opinion. Only 21% of respondents expressed an opinion about whether tagging was ethical. For newspaper readers (the *Daily Mail* and *Guardian*), this opinion was likely to be negative, whereas for *The Conversation* (where readership is more academic), opinion was more balanced. Public perception can impact conservation success, and affect funding, where this derives from taxation or donation. Though willingness to comment on online stories is low, reactions to this story expose important questions for scientists seeking to engage with, and convince, the public of the ethical and welfare aspects of their work.

## 1. Introduction

A variety of markers (e.g., leg rings and wing flags) and tracking devices (e.g., Global Positioning System (GPS) trackers) are routinely used in the study of free-living wild animals. They have become an important tool in global wildlife monitoring and species protection [1]. In many cases, these tags are not visible to humans outside the science and conservation world because of the remote settings in which the animals live. However, members of the public may come across tags on wild animals, either directly or via media sources, and may have an opinion about their use. They also, typically, pay for the research being performed.

Nguyen et al. (2012) investigated the opinions of fishers to biotelemetry, finding that most of the 68 respondents were positive or indifferent [2]. The small amount of negativity was based on potential stress during the gastric tagging procedure and possible impacts on migration. Other evidence of public opinion comes from direct encounters with researchers in the field. For example, in 2007, researchers in Australia were confronted with protests and subsequently legal challenges when using hot branding as a marker on elephant seals [3].

**Figure 1:**
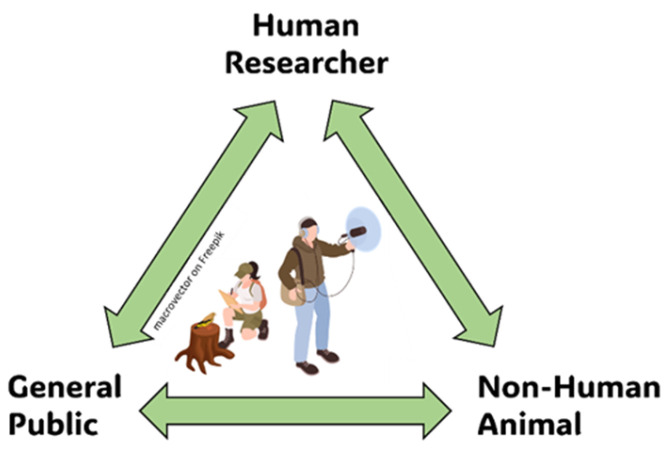
Interactions between public, researcher, and animal stakeholders in wildlife research.

Palmer and Greenhough [4] identify relationships with the public as a key challenge to successful research with wildlife. This echoes a survey of conservationists by Sandbrook et al. [5], where the third most agreed with statement (out of 38) was ‘Conservation will only be a success if it has broad public support.’ Researchers may encounter human stakeholders while in the field, posing potential risks to themselves, the science, and the welfare of the animals [4]. This paper focuses on the public perception of the relationship between academics and animal subjects. (Figure 1).

Researchers deploying tagging equipment must, at least in some countries, justify their use to an ethical review body, outlining the potential benefits and potential costs to the animals’ welfare. Part of this process can involve carrying out pilot studies to test equipment and techniques for capture and handling. One such project was undertaken on a small group of Australian Magpies (*Gymnorhina tibicen*)—with an unusual and intriguing outcome.

The study, carried out by Crampton et al., 2022, planned to trial a passive-release tag to see whether they could be removed without recapturing the birds [6]. A focal group of 10 birds was habituated to using a ground feeding station. Subsequently, five of those birds were captured and fitted with a GPS archival tag, attached by a harness. The birds were also fitted with an aluminum leg band. The harness was designed with a single weak point [6].

The first tracker, on a juvenile, was removed by an untagged female within an hour of attachment [6]. Meanwhile, a second bird was having their tracker removed by a different, untagged bird (ibid.). Over the following days, magpies were regularly spotted in the trapping area with leg bands, but no GPS tags were seen [6].

Alternative attachment methods are being considered that will be better tolerated by the birds [6]. The authors postulated that what happened may be an example of ‘rescue behavior’, as outlined by Nowbahari and Hollis [7], whereby a distressed individual is assisted, with no obvious benefit to the ‘rescuer’. It also indicates the magpies’ capacity for complex problem-solving.

The phenomenon was reported in the *Conversation* in February 2022, by one of the scientists involved [8], and subsequently picked up by other newspapers, including a humorous cartoon in the *Guardian* (First Dog on the Moon [9]). Readers of the *Conversation*, and some of the newspapers, were given the opportunity to comment on the story, which offers a limited, but useful, window into the views of a predominantly non-expert group of individuals.

The facility to comment on online news stories has been lauded for providing an opportunity for the public to share their opinions and for allowing a range of viewpoints and interpretations to be expressed [10]. The anonymity usually permitted may encourage more introverted readers, and atypical views, to be expressed [11].

Motivation to comment online has been categorized into four dimensions, describing the benefits users may receive from these interactions (‘gratifications’) [10] (Table 1). In a survey of online readers (commenters and non-commenters), Springer, Engelmann and Pfaffinger [10] found that social interactive motives drove users who made comments. Among those reading comments, cognitive and entertainment motives were found. Examining how readers responded to the case study here may provide insights into what drove them to comment and whether this relates to their views of tagging.

Although, as Larsson [12] notes, most readers of online news coverage do not post comments, those that do provide an insight into public reaction. These ‘below the line’ comments were explored to address the following questions: (1) How do readers respond to the tagging of magpies in the story or to wider issues around tagging? (2a) What is the overall balance of positive and negative sentiment among comments? (2b) Are the general public more in favor of tagging than a more academic readership?

## 2. Materials and Methods

### 2.1. Data Sources

I sought to balance political bias in readership by analyzing comments from left- and right-wing publications (see Table 2). The original article was published in the *Conversation*, which publishes articles by academics. The cartoon appeared in the *Guardian* online, a left-leaning UK broadsheet.

To balance these publications, I looked for an article in a right-wing newspaper, as ranked by AllSides.com (accessed 26 December 2022). The cartoon was published by a UK newspaper and the original article was accessed via the UK edition of the *Conversation*. Therefore, a UK publication was also sought for comparison. The *Daily Mail* online (‘Lean Right’ score of 4) was selected because access to comments was readily available. Other publications did not offer a comment section, or comments were not accessible for analysis.

Though the articles were accessed on the UK editions, the comments had a wide distribution, with many from Australians with first-hand experience of the species (based on self-reporting, idioms and cultural references). The *Conversation* has a stated aim to share academic knowledge (https://theConversation.com/au/audience accessed 25 October 2023). Its readership includes 18% ‘academic’, 15% ‘teachers’, 12% ‘medical/healthcare’, and 10% ‘government/policy’. Readership of the *Daily Mail* and *Guardian* may also include academics and those with conservation expertise, but with a more general news focus, many readers will be from the general population, with no specific expertise in this area.

### 2.2. Organizing the Data

The total number of reader comments available for analysis across all three articles was 680 (from 395 unique individuals). The *Conversation* included 77 comments, though 18 of these were from the author, clarifying points or justifying their work. Only reader comments were included in the analysis (hence *n* = 59, Table 2).

The *Guardian* chose to present the story in the form of a cartoon, in contrast to the other publications. This may have generated more interest and account for the larger number of comments on this site. Although there were 507 *Guardian* comments, from 253 individuals, 7 comments were removed by moderators, with 4 individuals not making any accepted comment. Because the content of their contributions is unknown, these comments/individuals were removed from the analysis (see Table 2).

The *Daily Mail* and the *Guardian* allowed ‘upticks’ as an expression of support for a particular comment. Whether the comment was isolated, or part of a thread, was also recorded.

Each user was allocated an anonymized ID number, as was each individual comment. This ensured that multiple comments by the same user could be separately identified and that replies were associated with the correct thread. The possibility that an individual may comment on more than one article, using a different username, must be acknowledged, though it seemed unlikely and certainly there were no occurrences of the same username being used across different publications.

### 2.3. Analytical Approach

This was a mixed-methods study, with comments analyzed quantitatively and qualitatively, using NVivo and MS Excel. A 6-step Reflexive Thematic Analysis, as outlined by Braun and Clarke [14,15], was used as a framework for the systematic exploration of the dataset. This involved data familiarization, initial inductive coding, development, the refinement and definition of themes, and relating findings to research questions. Thematic analysis was combined with an analysis of discursive elements, such as the expression of emotions and responses to others, using an interpretive approach [16]. To maintain anonymity, quotes are attributed using the comment ID number generated in NVivo. Although rarely appearing, emojis, emoticons and ‘text-speak’ abbreviations were used to assist in the interpretation of meaning.

To ascertain overall approval/disapproval of tagging, a ‘judgment’ rating was allocated to each user (see Table 3). Where a user posted more than once, only one ‘judgment’ code was recorded, taking into account all that person’s comments (there were no instances of readers changing their view in subsequent posts). Intercoder Reliability was assessed for judgment ratings because this variable involved a greater degree of interpretation and generalization than the qualitative analysis of themes [17]. A separate researcher coded 40 randomly selected commenters (https//www.random.org), using the 4 judgment codes in Table 3 [18]. This represented 10% of the total commenters [17]. Cohen’s Kappa for the 2 coders was 0.86, indicating a high level of agreement [17,18].

## 3. Results

A glance at the *Conversation* in Table 2 suggests that some users were posting in a thread containing one or more replies (37 separate users, contributing 59 comments). Forty-one of the fifty-nine total comments were replies to another post (69%). In the Daily Mail, this proportion was 18% and, in the *Guardian*, it was 63%.

In all three comment sections, most users posted only once or twice—even if as a reply to another’s post—rather than engaging in back-and-forth communication. In the *Conversation*, the comment space was dominated by one person who contributed 11 posts, including replies to other users. In the *Guardian*, one user, in particular, responded to replies to their original posts, suggesting they had accessed the online forum to engage with others.

### 3.1. Thematic Analysis

**Figure 2:**
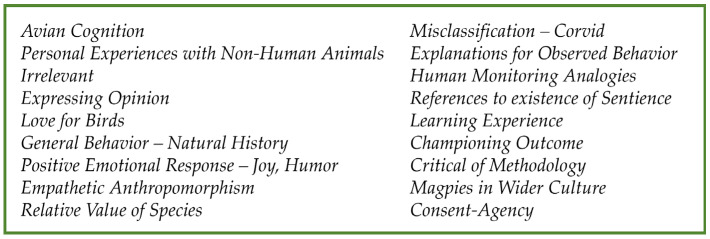
Frequently occurring references in the dataset.

Reference codes found on at least 20 occasions are listed in Figure 2. The full codebook is available in Supplementary Materials (S1). Over 100 of the comments were classed as ‘irrelevant’ (102 of 680, or 15%) and included comments not related to tagging, birds, or wildlife research ethics, as well as off-topic references, for example, IT issues.

When irrelevant comments are excluded, the most frequently occurring codes (see Figure 2) broadly overlap to form three main themes: (1) sharing personal feelings and experiences, (2) comparing the merits of different species, and (3) sharing knowledge and opinion.

In addition to the main themes, it was notable that some comments highlighted the learning potential of the research. Nine comments commended the scientist for sharing the experience, despite it having an unintended outcome. Most of these came from readers of the *Conversation*, with one such response in the *Guardian* comments. Thirteen comments referred to the value of the data, both for advancing research methodology and for understanding animal behavior. Some people also suggested some practical alterations to the equipment.

#### Main Themes

1. Sharing personal feelings and experiences

**Figure 3:**
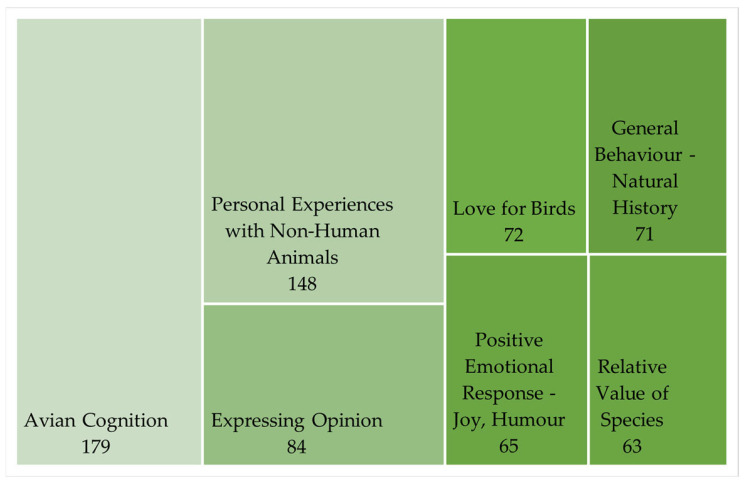
Codes occurring more than 50 times in the dataset (excluding irrelevant comments). Shading indicates the prevalence of each code.

Descriptions of personal experiences with non-human animals (chiefly birds) were found in 148 comments (Figure 3). Many of these were associated with positive feelings, and descriptions of positive impacts on the user’s wellbeing. Personal encounters with non-human animals were clearly important to commenters and were described as providing entertainment, companionship, and a sense of kinship.

The way in which some people described their encounters involved ascribing recognizable and shared characteristics to the individuals. Relating to the birds in these ways was an important part of the personal enjoyment and wellbeing that many users described. In this analysis, such comments were categorized as ‘empathetic anthropomorphism’ [19] and included references to sentience, personality, and intentionality among non-human animals. For example, the reader below talks of ‘a chorus of approval’, a clear intentionality and expression of emotion that may also be expected of human onlookers in a similar situation.

*“When a magpie became entangled in a “bird friendly” bird net encasing our favorite fruit tree, we slipped quietly under the net to try and free him/her. A small crowd of magpies gathered on the shed roof nearby and watched, making encouraging warbling noises. When freed there was—and there was no doubt about this—a chorus of approval and finally, a song of praise…”*

(Respondent 327)

Other emotional responses tended to reflect positive feelings (particularly humor) associated with reading the story or cartoon (see code book S1). These positive emotions were not directly related to magpies wearing tags, but rather the entertainment value of the reporting, particularly of the cartoon. This is a potential drawback when looking for readers’ responses in relation to the tagging story itself. Other expressions of emotion were uncommon, though there were comments expressing empathy, negative emotions, or general interest in the story. The reader below equates the animals’ behavior with how they themselves would behave, showing empathy for the animal subjects.

*“How would you like a tracker attached to you without consent. I believe I’d fight back.”*

(Respondent 283)

2. Comparing the merits of different species

The existence of cognitive abilities among birds, especially the magpies in this story, was found in 179 references (Figure 3). Commenters were keen to champion the magpies’ problem-solving abilities, with some users willing to compare avian intelligence unfavorably with that of humans. Acknowledging cognitive abilities in other animals can be a feature of anthropomorphism [19] but has been categorized separately because a) it was such a prominent topic in the dataset, and b) its occurrence is not always associated with ‘empathetic anthropomorphism’.

*“…They are such an intelligent bird—beats humans every time.”*

(Respondent 271)

*“This article appeared in the Conversation a few days ago, and caused quite a ruckus from an amazing number of people. The ruckus was not because of how amazing Magpies are, but how stupid scientists are…”*

(Respondent 435)

The role of magpies as pests was discussed in 37 comments. Much of this focused on the European magpie *Pica pica*, though some commenters shared stories of *G. tibicen* attacks on humans. The notion of *P. pica* as a pest was rebuffed by some, and others championed *G. tibicen* as a beautiful songbird, with which we should ‘peacefully coexist’.

*“For a group of [Australian] magpies, we are “their” humans… we don’t hassle them, they don’t hassle us. Peaceful co-existence.”*

(Respondent 393)

The tone and content of some comments reflected the divisive nature of certain species of birds, though specific pejorative language (e.g., ‘pests’, ‘evil’, ‘vermin’) was not much in evidence. It is difficult to ascertain how many users like or dislike magpies. Commenters were not asked specific questions, and there were many cases of misclassification (likely to be an underestimate because instances were only counted where the wrong species/family was clearly referenced). However, the relatively high number of references to the love of birds, and their intelligence (Figure 3), suggests that dislike was fairly low among responders.

Mentions of the existence of sentience among magpies and other animals were also found in the comments, as were comments championing the outcome of the story.

*“Well done. two fingers to the scientists until they design something that will really annoy them.”*

(Respondent 98)

*“Clearly these magpies were able to communicate that they didn’t like these sensors. Good on them for figuring a way to get rid of this intrusion. Researchers should take this reaction into consideration for future studies!”*

(Respondent 198)

Such comments provide further evidence of the value that most users placed upon the species and/or animals in general. Discussions on the relative value of different species overlap with the following theme (sharing opinion).

3. Sharing knowledge and opinion

A key motivation for posting a comment appeared to be the desire to share knowledge or opinions. Users were keen to share their knowledge of natural history, with 71 references among the comments. This knowledge tended to be shared respectfully, with the intention of adding to, or correcting, the information under discussion, and included links to examples (e.g., papers and footage of animal behavior research).

Forty-five comments discussed possible explanations for specific behaviors. There was some debate about whether the magpies in this case study were demonstrating altruism —behavior that benefits another, at a cost to themselves [20]—with alternative suggestions that they were drawn to shiny parts of the harness (probably from users mistaking the species for a corvid) or that they were expressing innate parasite-removing behavior. In the *Conversation*, the author replied to these comments, explaining why they considered altruism a possible explanation (the release point was tiny and required tenacity to find, and the rescue bird was not wearing a tracker).

*“They are incredibly intelligent and curious, and of course social. I do wonder though whether they were actually rescuing each other, or just being inquisitive about their tracker…”*

(Respondent 42)

*“Altruism can occur for sure! Rescue behavior is quite rarely observed, however.:)”*

(Respondent 47)

*“Surely putting a removable tracker on a social corvid that routinely allogrooms is asking for trouble?”*

(Respondent 73)

Opinions expressed about wildlife tagging (of which there were 84) may be based on a degree of expertise/research, on ‘gut-feeling’, empathy with other animals, views about the value of animals, or the value of individual species.

*“[…] It seems to also present a case of a somewhat round about negotiation of consent. When we put on trackers/flags/rings we try to make them as unobtrusive as possible but can’t tell exactly how annoying they are. These magpies have provided a great example of agency in removing the trackers. This brings new ethical considerations to the work…”*

(Respondent 1)

Some commenters expressed their criticism of the scientists by equating the animals’ responses to what would happen if the researchers were placed in a similar situation.

*“Who said the GPS trackers were non-invasive? The scientists, of course. Who, I am sure if they were imposed upon to wear trackers of a proportionately equivalent size to what they were sticking on the magpies, would do their damnedest to be rid of. A scientific survey of scientists seems long overdue. A collection of stick on and strap on devices used on birds and animals should be attached to a representative group of scientists and research over a lengthy period conducted…”*

(Respondent 613)

Of the 84 comments expressing an opinion, only 5 used personal anecdotes of encounters with animals to support their argument. This suggests, perhaps, that it is a distinct group, with a different motivation for contributing than those who are describing personal encounters with wildlife.

### 3.2. Balance of Opinion

In total, 84 (of 395 individual respondents, or 21%) respondents volunteered a clear opinion on the ethics of wildlife tagging (see Table 4). Of the remaining 79%, the comments either did not express an opinion, or the viewpoint would have required an interpretive leap that the short snippets of information did not really justify. These remaining 311 individuals were classified as ‘irrelevant’ and included comments that were unrelated to the specific ethics of animal tagging. These comments were included in the thematic analysis and were only considered irrelevant in terms of ethical judgment.

Of the 84 individuals expressing an opinion, 51 were critical of the use of tags (either in this particular case or on wildlife in general), 18 were ‘not wholly for or against’, and 15 wrote positively in support of tagging research. It was expected that the readership of each article would differ, so the subsequent analysis treats each article’s responses separately.

#### 3.2.1. Comparison of Opinions Between Publications

Almost half (49%) of contributors to the *Conversation* comment section expressed an opinion about the use of tags. This was far greater than either the *Daily Mail* (28%) or *Guardian* (14%). This could be a reflection of a more academic readership in the *Conversation*. It may also be because the article’s author responded to a number of posts, and users may have been hoping to engage directly with them. On visual inspection, opinion in the *Conversation* appeared more balanced (though there are insufficient data to establish significance) (Table 5). This result is not surprising given the different readerships.

While the sample sizes are small, there is a clear difference in positive and negative judgements among *Daily Mail* and *Guardian* responses (Table 5). Of those readers who expressed an opinion, that opinion was more likely to be negative than positive about the use of tags.

#### 3.2.2. Indirect Support

Upticks can be seen as an indirect measure of public opinion, where they are associated with ‘judgment’ comments. Table 5 shows the number of upticks only for those responses that expressed an opinion on tagging. Comments in the *Daily Mail* received far more upticks, in total, than those of the *Guardian*. There is a clear difference in support for positive and negative comments among *Daily Mail* responses. There appears to be a difference in the *Guardian* data, confirmed with a binomial test (Table 5). For both publications, readers were more likely to ‘vote’ for a negative judgment than a positive one. It must be noted here that independence cannot be guaranteed because it is possible that readers ‘upticked’ more than one comment. Additionally, these results are heavily skewed by individual comments with a large number of upticks. Two, in particular, were notable for making comparisons with human tracking technologies and it is possible that upticks represent agreement about that aspect, rather than non-human animal tracking. However, binomial tests where these dominant comments were excluded were still highly significant, suggesting that the outcome reflects genuine levels of support.

## 4. Discussion

This case study provides a snapshot of the interactions between some of the stakeholders in wildlife research (see Figure 1). Returning to research question 1 (how people respond to the tagging story), readers who commented on the articles took the opportunity to share personal encounters with non-human animals, to give their opinions about the tagging story, and to simply express admiration for the birds (in particular, their cognitive abilities). These comments could be couched in empathetic language. Harrison and Hall [21] state that our tendency to ascribe humanness to non-human animals in this way fulfills an important social need.

The category ‘empathetic anthropomorphism’ [19] was used in this analysis as a distinct type of anthropomorphism. Anthropomorphism has traditionally been used as a label for the (mis)attribution of human traits [19] and has acquired negative overtones. It has been seen as neglecting the uniqueness of animal species by considering them only in relation to humans [22]. Its use is broad, including anthropomorphic representations (e.g., animals in clothes) and attributing ‘human’ traits to animals that a person does not really believe they possess [19].

Egomorphism, coined by Milton [23], is a sub-category of anthropomorphism and describes perceiving non-human animals as ‘like me’, rather than ‘like a human’. This represents a significant move away from earlier interpretations that were both anthropomorphic and anthropocentric [22]. Empathetic anthropomorphism relates to others rather than only the self (egomorphism). It allows for the capability of humans to empathize with other individuals, even where the experience may be unlike their own. It has been suggested that empathetic anthropomorphism could be deliberately exploited to aid conservation [19].

Despite evidence for empathy, there were also examples of speciesism [24] among responses, with many users singling out species or groups of species as being more worthy of protection and admiration than others. This is perhaps evident in the scientists too. It is notable that the researchers say that they will not use this particular harness again on magpies, though they still advocate the development of magnetic-release trackers and the wider principle of tracking of wild animals [6]. The outcome of this pilot study may have been very different had the species (or those individuals) not been able to demonstrate their dislike of the tags.

The proportion of readers who are willing to comment on an online news story is low [12], so it is probable that commenters on the magpie story represent a small percentage of the total readership of each article. When examining the balance of opinion on the use of tags (research question 2), it is important to acknowledge that this is based on an even smaller subset and that the categorization of their opinion requires interpretation because it was not directly asked of them. Individuals who expressed views both in favor and against tag use may have been seeking clarity in their own opinion. However, readers who were indifferent may not have been sufficiently motivated to comment at all.

Unwillingness to participate in comment sections is particularly the case where the reader is concerned their view may be in the minority [11,25,26]. However, Nekmat and Gonzenbach [26] also suggest that, in some cases, readers may choose to not contribute a response if their opinion was already supported among the messages. Contributors to the *Conversation* showed a fairly balanced opinion on tag use and, while there is a majority view against tag use in the newspaper articles, even here, other viewpoints were expressed. It is interesting to note the headlines that the newspapers used, including the terms ‘cheeky’ (*Daily Mail*), ‘courageous’ and ‘bastards’ (*Guardian*), which may also have influenced readers.

These factors limit generalizability from the corpus to the wider readership, or to the general population, in terms of the predominant viewpoints. However, the comments may still reflect the range of perspectives that exist [27]. Those individuals who did post comments must have been motivated in some way. Looking at the content of user comments on the magpie story, all four of Springer, Engelmann and Pfaffinger’s dimensions [10] appear to be represented, with the desire to share information and educate (the cognitive dimension) being particularly prominent. The analysis here is based on free-text online comments, rather than specific survey data where people are asked about their motivations, so it is not possible to confirm this hypothesis. However, this motivation was shared with survey data from Kangaspunta [28] who concluded that one of the top three preferred reasons for commenting was correcting information (along with the perceived importance of the topic and stating personal opinions).

Kangaspunta [28] found that ‘provoking and entertainment’ were the least preferred motives for commenting on online news. Comments praising the humor in the magpie story and, particularly, the cartoon, suggest an entertainment motivation. Indeed, it is significant that many readers responded positively to the humor in the *Guardian* cartoon, and this makes it more difficult to compare with the text-only reports. The prevalence of personal tales of interactions with non-human animals also suggests an entertainment motivation among some users. However, there was very little provocation or debate. Given the relatively low number of people expressing a specific opinion on tagging, the personal identity dimension may be less of a driver.

The social integrative dimension as a motivator is supported by research into the personality types of those commenting online. Contrary to their expectations, Wu and Atkin [11] found that more introverted, less conscientious, or less open-minded individuals were more likely to comment in the pursuit of social recognition. Their data suggested that ‘agreeableness’ and ‘narcissism’ were the main personality predictors of motivations to comment on online news. The motivations of ‘agreeable’ contributors included sharing information and inviting opinion, which is in line with the results found here. The comment sections here tended to be agreeable in nature, involving making shared connections and informing and educating (aligning with both the social and cognitive dimensions of Springer, Engelmann and Pfaffinger’s motivation model [10]). ‘Click Speech’ (in this case, upticks) can reinforce a sense of social approval for the commenter [29].

The subject matter in this case may lend itself to more agreeable, ‘others-oriented’ (p. 74, [11]), and less narcissistic individuals. The story is not directly challenging people’s opinions on a controversial topic and may prompt readers to think about their own personal encounters with non-human animals. While this leads to a less confrontational exchange, the extent to which comments reflect wider opinion is more questionable. Those who are less agreeable, and who do not share the prevailing opinion, may be unlikely to contribute and thus hold an under-represented opinion on the subject.

The free-text nature of the online comment format does not require users to commit to a particular position on the use of tags. This, in itself, provides an interesting insight because users must choose to contribute an opinion over competing motivations to comment.

In places, the discussion was often quite nuanced, showing an appreciation of the difficult decisions involved in modern scientific endeavor (ethical balance, human resources, funding, etc.). Despite this, the newspaper comments and upticks were overwhelmingly negative about the use of the trackers. Commenters on the *Conversation* article displayed more balance in their opinions towards trackers, perhaps reflecting the more academic readership. The caveat, that commenters may be biased towards an established predominant opinion, appears not to exert such influence upon readers of the *Conversation*.

In all three publications, there were some negative comments aimed at the scientists, illustrating why there may be a reluctance to publish negative outcomes, especially in popular, non-specialist outlets. In this case study, the author and their research team decided to share their experiences in a self-penned article, as well as providing interviews with journalists. This required a considerable investment of time and exposed the researchers to potentially negative reactions (albeit not face to face in the field). Some commenters acknowledged this effort and commended the scientists for being open about the experience. Engagement by the author helped to answer questions and allay people’s concerns, providing a useful insight into the information researchers may need to include in communications with the public, e.g., clarifying any impacts of tags on animal behavior, stating the ethical protocols of the research institution or government.

Writing in non-specialist publications represents one way for scientists to communicate what is happening in the field. While this case study was not a structured collaboration, it offers a hint at how technology could assist in reaching out to the public, much more broadly than is possible via ethics committees or local education inputs. The evolution of more interactive means of communication provides opportunities for more two-way discussions between scientists and the public, as advocated by Norton [30]. More interaction between scientists and commenters, while time-consuming, may be particularly well-received by those users who are motivated by social integrative gratification [10].

In resolving issues of environmental ethics, Norton [30] advocates public deliberation as a more pragmatic approach than traditional public education. Decisions are derived through open discussion and can be revisited as more is discovered or priorities change. In this way, individuals feel genuinely listened to, and those who disagree are not left behind. Harrison and Loring [31] point out that people are more likely to accept conservation decisions if they feel the process was equitable, even if they disagree.

Keulartz [32] suggests that a democratic deliberation of the form proposed by Norton might be too optimistic where there is significant value pluralism. An important distinction to consider is that of value disagreements and factual disagreements. This is where the expertise of the scientists plays a crucial role. Once parties are aware of the current state of knowledge (in this case, about tagging-related welfare), and the stated purpose of the research, the debate is distilled to differences in values, at which point, no single viewpoint necessarily overrules another.

These differences may be intractable and may come up against practical difficulties in allocating sufficient time to fully explore and resolve for every tagging project. Building good working relationships between stakeholders requires a significant investment in time. Perceived, or actual, status asymmetry between scientists and others can present significant challenges to open discussions about animal ethics [33,34]. Where formal ethics committees exist, feeling isolated or outnumbered may present a problem for the layperson [34]. More informal and anonymous communications may overcome this, although thorough engagement through discussion is more limited.

## 5. Conclusions

This was a small, opportunistic study where users were not specifically asked to give an opinion. The main motivations for commenters in this case study appear to have been to share stories and knowledge. Researchers would need to ask specific questions and/or provoke readers directly to ascertain their opinion more accurately. However, the data here illustrate some of the public perceptions of wildlife tagging, which they are not usually asked about directly. Larger studies, involving direct questioning, would be beneficial in uncovering public understanding and opinions of tagging, as well as their views on opportunities and methods for interacting with scientists.

Given the low numbers of people who comment on online news [12], the data presented here cannot be assumed to represent wider public opinion. Nevertheless, there was a clear negative reaction among many readers. In this case study, there appears to be agreement between the scientists and readers that the tags should not be used again, though there are important unanswered questions. To what extent was this agreement because of the way the case study was articulated (leading readers to that conclusion) and to what extent down to the animal behavior itself, as observed by the researchers? It is significant that these individual animals were able to express themselves so clearly. In a sense, they became participants in the ethical discussion that would normally involve only humans. What about where the animal behavior is less obvious or where there is more significant disagreement between human stakeholders?

Ethical review processes are dominated by academics, with a range of expertise, to discuss the issues. They have minimal public representation (and no animal representation). What is common practice in academia may be less acceptable to the people paying for the research. To reduce the likelihood of hostile confrontations with more vocal members of the public while researchers are conducting fieldwork, it is in their interests to engage with a wide pool of value judgements, including those expressed by non-specialists. Reporting and interacting in ‘non-traditional’ formats (i.e., cartoon, click speech, social media) may provide a means of reaching the wider public so that there is better mutual understanding and engagement.

## Figures and Tables

**Table 1:** Four dimensions of motivation to comment online (summarized from Springer, Engelmann and Pfaffinger [10]).

Cognitive Dimension	Satisfaction is derived from engagement in discussion, sharing information, answering questions and educating.
Entertainment Dimension	Commenting is a relaxing, diverting activity. It provides an opportunity to use humor and cope with emotions (which can lead to offensive comments).
Social Integrative Dimension	Commenting allows for social interaction via follow-up communication (though interactivity is usually low).
Personal Identity Dimension	Individuals are motivated to present their opinions. They enjoy recognition and validation from others.

**Table 2:** Overview of data sources used in this analysis. (* Total contributions accepted by moderators).

Publication	Leaning (From AllSides.com, as of 2022)	Data Source	Date Published	Total Reader Comments	Total Individual Commenters
*The Conversation*	Ranked ‘Lean Left’ but with low confidence	*Altruism in birds? Magpies have outwitted scientists by helping each other remove tracking devices.* [8]	21 February 2022	59	37
*Daily Mail Online*	Lean Right	*Cheeky magpies help each other remove sophisticated GPS harnesses–ruining a year of scientific work* [13]	22 February 2022	121	109
*Guardian Online*	Lean Left	*Magpies-courageous-heroes-or-little-feathery-bastards* First Dog on the Moon [9]	25 February 2022	500 *	249 *

**Table 3:** Code descriptors for ‘judgment’ codes.

Opinion	Description
Positive	Supportive of use of tags in wildlife research, or in this specific case
Not Wholly For or Against	Comment includes points in favor and against
Negative	Against tag use, or does not support use of tags in this specific case
Irrelevant	Comment does not make a judgment about the use of tags

**Table 4:** Examples from each ‘judgment’ code.

Opinion	Example from the Online Comments
Positive	*“To be fair, it’s only trying to understand them a bit better and further our knowledge about this fascinating bird. The trackers would have been removed anyway after the experiment.”* (Respondent 101)
Not wholly for or against	*“Thank you…a really interesting article illustrating researcher ethical responses to evidence. You raise important questions for philosophers, scientists and A(nimal) E(xperiment) C(ommittees) about emotion, empathy, altruism and consent in humans and non-humans—bring on the nanotech-trackers!”* (Respondent 64)
Negative	*“It shows that they definitely didn’t like the equipment, which goes against any assurances that such experiments do not harm or cause discomfort to the subjects.”* (Respondent 82)

**Table 5:** An analysis of comments classified as making a judgment on the use of tags. Upticks were not an option for *Conversation* readers. * Significant at *p* < 0.01; ** Significant at *p* < 0.001. Significance is based on a binomial test *p* = 0.5, assuming a random chance of an opinion being positive (*Daily Mail n* = 25; *Guardian n* = 31) or an uptick on a positive comment (*Daily Mail n* = 988; *Guardian n* = 429).

	*Conversation*	*Daily Mail*	*Guardian*
Total individuals making ‘judgment’ comments	18	30	36
Users making ‘judgements’ as % of total users	49%	28%	14%
Positive judgment comments	4	3	8
Negative judgment comments	6	22 **	23 *
Judgment comments not wholly for or against	8	5	5
Upticks for positive judgment comments	-	33	178
Upticks for negative judgment comments	-	955 **	251 **
Upticks for judgements not wholly for or against	-	268	43

## Data Availability

The codebook generated in this analysis is available in the Supplementary Materials (S1). The original data presented in the study are openly available at *The Conversation*, “Altruism in birds? Magpies have outwitted scientists by helping each other remove tracking devices”, the *Daily Mail*, “Cheeky magpies help each other remove sophisticated GPS harnesses—ruining a year of scientific work” and the *Guardian*, “Magpies: courageous heroes or little feathery bastards?”|First Dog on the Moon|The *Guardian*.

## Supplementary Materials

The following supporting information can be downloaded at https://www.mdpi.com/article/10.3390/ani15142053/s1, Table S1: codebook.
