# Supplementary material for: Reader Responses to Online Reporting of Tagged Bird Behavior
Source: Animals (Basel). 2025 Jul 11;15(14):2053. doi: 10.3390/ani15142053 (PMC12291723; doi:10.3390/ani15142053)
Supplement: Supplementary file 1 [file animals-15-02053-s001.zip › animals-3718237-supplementary.pdf]

Table S1 Complete Codebook

Where original coding has been amalgamated during the secondary coding process, user comments are unique. So, comments are not counted twice within a theme, *unless* parts of a comment have been coded separately or the user is clearly making distinct points.

For reasons of succinctness, sub-categories and examples are only shown for those codes with over 50 references among the comments.

**Code** *(sub-category)*		**References**	**Description**	**Examples***
**Avian Cognition**<br>*Ascribes intelligence to birds*<br>*Comparing avian and human problem-solving*<br>*Disputing traditional use of 'bird brain'*<br>*More intelligent than scientists*	 *136*<br>*19*<br>*9*<br>*15*	**179**	Mentions of intelligence among magpies or other bird species; includes comments disputing traditional use of 'bird brain'	*Not so bird brained after all, cleverer than people [181]*
**Personal Experiences with Non-Human Animals**<br>*Anecdote from other source*<br>*General human-nonhuman animal interactions*<br>*Personal stories*<br>*Response to a personal story*	 *2*<br>*16*<br>*100*<br>*30*	**148**	Sharing anecdotes of personal experiences of human-non-human interactions	*… these magpies and my family became accustomed to each other(...) As my daughter was growing up she could also play with them and it appeared the senior magpies would look after her… [77]*
**Irrelevant**		**102**	Comments not related to the story/tagging/birds e.g. off-topic politics, IT issues/comments; includes removed comments	*lol...always leave home without a mobile phone! Love it! Nah...at my age, I'll carry one. I might wander off and get lost otherwise. [286]*
**Expressing Opinion**<br>*Judgement – Negative*<br>*Judgement – Not wholly for/against*<br>*Judgement – Positive*	 *51*<br>*18*<br>*15*	**84**	All ‘Judgement’ codes expressing an opinion about the use of tagging in wildlife research	*… tag on storks’ leg caused the poop to accumulate underneath (…) and cause damage. On the other hand (…) the [swan] collar provided valuable (…) information and apparently did not affect the bird that much [72]*
**Love for birds**<br>*Love for Australian magpies*<br>*Love for Corvids*<br>*Love of birds*	 *54*<br>*8*<br>*10*	**72**	Love for birds; includes codes for love of Australian magpies, corvids, other birds	*I love these birds. So clever and they have a playful spirit. [194]*
**General behaviour - natural history**		**71**	Sharing knowledge of natural history/wild animal behaviour	*Australian magpies aren't like that. They live in families and they look after each other. [474]*
**Positive Emotional Response - Joy, Humour**		**65**	Positive feelings associated with the story itself; enjoyment from reading	*That is so funny! I laughed at every frame. Well done doggie - and GO Magpies... [271]*
**Empathetic Anthropomorphism**		**64**	Attributes sentience/motivation/intentionality to non-human animals	*I've seen them grieve, though. ... And groom each other, and yawn, and look angry ...[675]*
**Relative Value of Species**<br>*Pests-Debate*<br>*Ranking species*<br>*Magpies Divisive Species*	 *37*<br>*14*<br>*12*	**63**	Ranking opinion of species; speciesism; magpies as pests or valued songbirds	*Train them to attack Indian Mynahs! [253]*<br>*I like magpies except in spring when I hate the buggers. [627]*

Code	References	Description
Misclassification-Corvid	49	Comments where misidentification of species is clear or those correcting this error
Explanations for Observed Behaviour	45	Discussion of altruism in non-human animals; debating whether this story is evidence of true altruism; Suggests other possible reasons for bird behaviour shown in this study
Human Monitoring Analogies	42	Comparing non-human animal tagging with human monitoring technologies; includes discussions by those for and against such monitoring
Learning Experience	38	Acknowledges scientists' attempts to improve devices, minimise impact; Commends scientist for sharing the story despite the unanticipated, negative outcome; Importance of sharing information; The data is valuable even though it was not the intended outcome
Championing Outcome	35	Applauding the work of the magpies in the story
Critical of methodology	30	Comments criticising methodological approach; Concern about impact of tags affecting science outcomes
Magpies in Wider Culture	30	Literature, TV, film, folktales and superstitions (includes corvid references)
Consent-Agency	26	Comments advocating or questioning animal consent
Receives response from author	15	Comments and questions receiving a response from the scientist who authored the Conversation article
Championing science	15	Supportive of scientific pursuit of knowledge
Emotional Response - Empathy	14	Describing own feelings of what wearing a device would be like
Projecting human societal behaviours	14	Projecting human scenarios onto magpies (i.e. situations/behaviours in human society) e.g. taking part in tracking experiments, betting, human parliaments.
Research Ethics	13	Discussion of research dilemmas, balance of ethics; ethics of ‘interference’, ‘respectful’ treatment
Links to other studies or video of similar animal behaviour	13	Links or reference made to other studies of tagging effects or similar behaviours
Negative Emotional Response	12	Negative emotional impacts of the story such as anger, sadness
Queries	12	Seeking further details from author; Stating questions/hypotheses for further study
Positive Emotional Response - Interest, Fascination	11	Emotional responses that mention fascination with subject matter
Power dynamics - control	10	Questioning dynamic between researcher and subject animal; describes balance of power or desire for dominance
Dislike of magpies (unspecified)	10	Expresses dislike of magpies (species could be estimated by content)
Animal Rights	9	Mentions animal ‘rights’ or uses rights language i.e. moral absolutism, ethical imperatives
Peaceful Coexistence	8	Advocates concept of peaceful coexistence
Discussion of role of science in ecological damage	8	Blaming science itself for causing some ecological damage

Critical of funding or research focus	7	Feel funding should be diverted elsewhere
Consciousness	7	Specific references to animal consciousness
General animal cognition	7	References to cognition in non-avian species
Cruelty	6	State opinion that the study was cruel - words may include cruelty, suffering, abuse
Sarcasm	6	Comment uses sarcastic tone
Habitat Destruction - Species Loss	6	Emphasises overriding importance of habitat loss/species loss
Alternative Methods	6	References to role of observational methods or other alternatives to tags
Avian Cognition - Lack of	5	Suggesting birds lack cognitive capacity
Lack of empathy shown by scientists	5	Scientists not ‘embodying’ animal subjects
Sceptical of claims of no harm	4	Distrust of claims that tagging does no harm
Avoiding anthropomorphism	3	Anxious to avoid accusations of anthropomorphism in interpreting behaviour
Competitive nature of science	3	Possible negative impacts of nature of modern science on work, i.e. desire for funding, scientists’ reputations
Role for social science in non-human animal science	2	Advocates role for social science
Tracking necessary	2	Describes need for tracking to better understand nature/to protect species
Intrinsic Value	2	States that animals have intrinsic value
Other studies of intraspecific interactions	1	Provides links/ref to other studies of intraspecific interactions
Magpie Captivity	1	Refers to magpies in captivity; captive studies
Pro-wildlife behaviour (human)	1	Advocates specific pro-wildlife actions that can be undertaken
Author was selective in responding	1	Claims that author in the Conversation only responded to certain comments

** Data obtained from online comments to the following articles:*

Potvin, D. Altruism in birds? Magpies have outwitted scientists by helping each other remove tracking devices. *The Conversation*. **2022** *February 21* https://theConversation.com/altruism-in-birds-magpies-have-outwitted-scientists-by-helping-each-other-remove-tracking-devices-175246 (accessed 27/02/22).

First Dog on the Moon. Magpies: Courageous heroes or little feathery bastards. *The Guardian* https://www.theGuardian.com/commentisfree/2022/feb/25/magpies-courageous-heroes-or-little-feathery-bastards (accessed 26/02/22).

Ferrier, T. Cheeky magpies help each other remove sophisticated GPS harnesses – ruining a year of scientific work. *Daily Mail/Australian Associated Press* **2022** *February 22* https://www.dailymail.co.uk/news/article-10537623/Cheeky-magpies-help-remove-GPS-harnesses-ruining-year-scientific-work.html (accessed 22/02/22).
